# Supplementary material for: Complexome profiling of the Chlamydomonas psb28 mutant reveals TEF5 as an early PSII assembly factor
Source: Plant Cell. 2025 Mar 17;37(6):koaf055. doi: 10.1093/plcell/koaf055 (PMC12164590; doi:10.1093/plcell/koaf055)
Supplement: koaf055_Supplementary_Data [file koaf055_supplementary_data.zip › psb28_interactive.zip!/plots/intensity/qualityPlots/peptides/_Cre07.g323600.t1.1.html]

### Cre07.g323600.t1.1

Trivial name:   
SeqCoverage [median]: 11.60  
Mass: 21.03 kDa  
  
 

| Sequence | MissedCleavage | Mass [Da] | wtIntensity | muIntensity | Score |
| --- | --- | --- | --- | --- | --- |
| AGQESENVIVGGR | 0 | 1314.65 | 13143.67 | 13348.40 | 223.07 |
| QLYEAMVER | 0 | 1137.55 | 0.00 | 776.97 | 87.64 |
|  |  |  | 13143.7 | 14125.4 |  |
